# Health and Performance Challenges in the Era of Human Enhancement: Insights from Sport Medicine Professionals

**DOI:** 10.1007/s40279-025-02258-7

**Published:** 2025-06-16

**Authors:** Ke Hu, Christian Schneider, Mark R. Hutchinson, Emin Ergen, Michael Geistlinger, Iain Killoughery, Klaus-Michael Braumann, José Kawazoe Lazzoli, Jane Seto, Xavier Bigard, André Debruyne, Anna Jegier, Theodora Papadopoulou, Pedro Manonelles, Francisco Arroyo, Mourad Ghrairi, Ana V. Cintrón, Petra Zupet, Marcelo Bichels Leitão, Umutcan Kayikci, Daniel Arkader Kopile, Fabio Pigozzi, Chiara Fossati, Alessia Di Gianfrancesco, Luigi Di Luigi, Kirill Micallef Stafrace, Anca Ionescu, Bernd Wolfarth, Metin Ergün, William O. Roberts, Konstantinos Natsis, Camille Tooth, Sandra Rozenštoka, José Antonio Casajús, Borja Muniz-Pardos, Roberto Lohn Nahon, Malav Shroff, Minhao Xie, Demitri Constantinou, Dina CJanse van Rensburg, Bülent Ülkar, Andrew Jowett, Victoriya Badtieva, Jean-François Kaux, Peter Baumgartl, Clea Hadjistephanou Papaellina, Jürgen Steinacker, Julio Motta-Pensabene, Rüdiger Reer, Norbert Bachl, Sergio Migliorini, Maher Zahar, Mark Stuart, James Bilzon, Giuseppe Massazza, Bruno Di Pietro, Khaled Massoud Hassan, Felix Albuquerque Drummond, Bertrand Fincoeur, Andrea Petróczi, Yannis Pitsiladis

**Affiliations:** 1 Department of Sports and Health Sciences, Hong Kong Baptist University, Hong Kong SAR, China; 2 Orthopaedic Centre Theresie, Munich, Germany; 3 Orthopaedics and Sports Medicine, University of Illinois at Chicago, Chicago, USA; 4 Gloria Sports Arena, Sports Medicine Unit, Belek, Antalya, Turkey; 5 Department of International and European Law and Fundaments of Law, University of Salzburg, Salzburg, Austria; 6 Department of Sport and Exercise Medicine, Faculty of Psychology and Human Movement Science, University of Hamburg, Hamburg, Germany; 7 Biomedical Institute, Fluminense Federal University Medical School, Niterói, Brazil; 8 Murdoch Children’s Research Institute, Parkville, Australia; 9 University of Melbourne, Parkville, Australia; 10 Union Cycliste Internationale, Aigle, Switzerland; 11 European Federation of Sports Medicine Associations (EFSMA), Lausanne, Switzerland; 12 Department of Sports Medicine, Medical University of Lodz, Lodz, Poland; 13 Defence Medical Rehabilitation Centre (DMRC), Loughborough, United Kingdom; 14 Department of Sports Medicine, San Antonio Catholic University of Murcia, Murcia, Spain; 15 Sport Med, Fédération Internationale de Football Association (FIFA) Medical Centre of Excellence, Zapopan, Jalisco Mexico; 16 Department of Sports Medicine, FIFA Medical Centre of Excellence, Dubai, United Arab Emirates; 17 Puerto Rico Sports Medicine Federation, San Juan, Puerto Rico; 18 Department of Physical Medicine, Rehabilitation and Sports Medicine, School of Medicine, University of Puerto Rico, San Juan, Puerto Rico; 19 Institute for Medicine and Sports (IMŠ), Ljubljana, Slovenia; 20 Instituto de Endocrinologia E Medicina Do Exercico, Curitiba, Brazil; 21 Division of Maternal Fetal Medicine, Department of Obstetrics and Gynaecology, Faculty of Medicine, Hacettepe University, Ankara, Turkey; 22 Antidoping, Doping Studies and Analysis of Antidoping Policies Master Programme, Paris Nanterre University, Paris, France; 23 Olympic Studies Center, German Sports University, Cologne, Germany; 24 Cardiac Rehabilitation Department, National Institute of Cardiology, Health Ministry, Laranjeiras, Brazil; 25 Department of Movement, Human and Health Sciences, University of Rome Foro Italico, Rome, Italy; 26 Mater Dei Hospital Malta, Triagon Academy, Marsa, Malta; 27 Authority for Integrity in Maltese Sports (AIMS), Marsa, Malta; 28 National Institute of Sports Medicine, Bucharest, Romania; 29 Discipline of Sports Medicine, Carol Davila University of Medicine and Pharmacy, Bucharest, Romania; 30 Universität Berlin and Humboldt-Universität, Berlin, Germany; 31 German Society for Sports Medicine and Prevention, Deutsche Gesellschaft für Sportmedizin und Prävention (DGSP), Frankfurt, Germany; 32 Turkish Sports Medicine Association, Izmir, Türkiye; 33 Department of Family Medicine and Community Health, University of Minnesota, Minneapolis, United States of America; 34 Department of Anatomy, School of Medicine, Faculty of Health Sciences, Aristotle University of Thessaloniki, Thessaloniki, Greece; 35 Physical Medicine and Rehabilitation, University Hospital of Liege, University of Liege, Liege, Belgium; 36 French-Speaking Olympic Sports Medicine Research Network (ReFORM) International Olympic Committee (IOC) Research Centre for Prevention of Injury and Protection of Athlete Health, Liege, Belgium; 37 Riga Stradins University, Riga, Latvia; 38 Sports Laboratory, LLC–International Federation of Sports Medicine (FIMS) Collaborating Centres of Sports Medicine (CCSM), Riga, Latvia; 39 Latvian Sports Medicine Association, Riga, Latvia; 40 Physiopathology of Obesity and Nutrition Networking Biomedical Research Centre (CIBEROBN), Carlos III Health Institute, Madrid, Spain; 41 Department of Physiatry and Nursing, Faculty of Health and Sport Science (FCSD), University of Zaragoza, Zaragoza, Spain; 42 Exercise is Medicine Spain, University of Zaragoza, Zaragoza, Spain; 43 Growth, Exercise, Nutrition and Development (EXER-GENUD) Research Group, Faculty of Health and Sport Sciences, University of Zaragoza, Zaragoza, Spain; 44 Titular Member of the Brazilian Society of Exercise and Sports Medicine (SBME), São Paulo, Brazil; 45 World Olympians Association, Lausanne, Switzerland; 46 China National Institute of Sports Medicine (NISM) Sports Medicine Hospital of NISM, Beijing, China; 47 Department of Exercise Science and Sports Medicine, Faculty of Health Sciences, University of the Witwatersrand, Johannesburg, South Africa; 48 South African Sports Medicine Association (SASMA), Pretoria, South Africa; 49 Section Sports Medicine, Faculty of Health Sciences, University of Pretoria, Pretoria, South Africa; 50 Department of Sports Medicine, Faculty of Medicine, Ankara University, Ankara, Turkey; 51 Olympic Park Sports Medicine Centre, Melbourne, Australia; 52 Sport Medicine, I M Sechenov First Moscow State Medical University, Moscow, Russia; 53 Sport Medicine, Moscow Center of Research and Practice in Medical Rehabilitation, Restorative and Sports Medicine, Moscow, Russia; 54 SportS2, University and University Hospital of Liège, Liège, Belgium; 55 Austrian Society of Sports Medicine and Prevention, Vienna, Austria; 56 Department of Education, University of Cyprus, Nicosia, Cyprus; 57 Institute of Rehabilitation Medicine Research, Ulm University, Ulm, Germany; 58 European Initiative for Exercise in Medicine (EIEIM), Ulm, Germany; 59 SportsMed Clinic Universidad de San Carlos de Guatemala, Guatemala City, Guatemala; 60 Austrian Institute of Sports Medicine, Vienna, Austria; 61 Medical Committee, World Triathlon (WT), Lausanne, Switzerland; 62 Tunisian Centre of Sports Medicine and Science, Oral Health Unit, Tunis, Tunisia; 63 Centre for Metabolism and Inflammation, Division of Medicine, University College London, London, United Kingdom; 64 Department for Health, University of Bath, Bath, United Kingdom; 65 British Association of Sport and Exercise Medicine, Doncaster, United Kingdom; 66 Division of Physical Medicine and Rehabilitation, Department of Surgical Sciences, University of Turin, Turin, Italy; 67 Sapienza University of Rome, Rome, Italy; 68 Egyptian Ministry of Youth and Sports, Mohandeseen, Giza, Egypt; 69 Egyptian Association of Sports Medicine, Cairo, Egypt; 70 Instituto de Medicina do Esporte (IME), Porto Alegre/Sports Medicine Institute, Porto Alegre, Brazil; 71 College of Humanities, Swiss Federal Technology Institute of Lausanne (EPFL), Lausanne, Switzerland; 72 Faculty of Health and Sport Sciences, Széchenyi István University, Győr, Hungary

## Abstract

**Background:**

In the pursuit of sporting success, some elite athletes prioritise peak performance over long-term health, frequently resulting in significant and enduring health consequences. The Enhanced Games (TEG) position themselves as a bold experiment in transhumanism, advocating for the use of performance-enhancing drugs (PEDs), including methods banned by World Anti-Doping Agency (WADA), to push the boundaries of human athletic potential.

**Objectives:**

The aim of this study is to explore the perspectives of sport physicians, sport scientists, physiotherapists and other allied healthcare professionals on treating and supporting “enhanced athletes”, with the view of informing future guidelines.

**Methods:**

Participants were invited via email and personal contacts within sport medicine communities to complete a brief anonymous survey via QuestionPro™. Descriptive statistics were performed using Excel™ and RStudio™.

**Results:**

A total of 323 healthcare professionals responded (82% were sport physicians), among whom 74% expressed a willingness to treat acute lesions and/or chronic diseases in “enhanced athletes”. In comparison, a considerable minority (30%) expressed support for assisting athletes in their use of PEDs and methods under medically supervised conditions, with high consistency across professional roles. A relatively high readiness was observed in sport physicians treating acute (77% versus 58%; *p* < 0.01) and chronic (75% versus 63%; *p* = 0.11) diseases for “enhanced athletes”. As far as WADA rules and/or national anti-doping laws apply, this support presupposes compliance with the code and the respective national laws to protect physicians from serious professional, legal and personal consequences.

**Conclusion:**

The preliminary findings align with the broader goal of fostering a sport culture that values both peak performance and the short- and long-term health of all participants. These results emphasise the necessity of implementing professional guidelines and comprehensive support systems designed to safeguard the long-term well-being of all athletes and underscore the urgent need for further research into the impact of TEG on sport and its community.

## Introduction

Against the backdrop of longstanding critiques of anti-doping policies [1], advocacy for permitting doping in sport [2], and the criminalisation of prohibited substances and methods by some countries such as Austria, France and Italy [3], The Enhanced Games (TEG) take a radical approach by fully embracing the use of performance-enhancing drugs (PEDs) and methods as a means to explore the boundaries of biological constraints and accelerate the current human form, which is described as transhumanism. Recently, TEG received Make America Great Again (MAGA)-inspired funding from Donald Trump Jr., who believes TEG represents “real competition and real freedom”, as well as possible political backing from President Donald Trump [4]. Dr. Aron D’Souza, president of TEG, urges the sport community to “trust the science and recognise that the Olympics will never be clean until there is an Enhanced Games”. He also promised that all anonymised data will be shared with the anti-doping community to help with the development of more effective testing methods [5]. This portrayal of TEG as a prequel to making sporting competitions truly fair, progressive and safe through full transparency raises several key questions: What kind of PEDs and methods will be used? How would such an initiative fit with national and international legislations for controlled substances? What safeguarding measures will be in place—and when and where—to protect athletes’ health? Who will oversee, administer and take responsibility for using PEDs and methods? How will potential addictions and complications from PEDs and performance-enhancing (PE) methods use be handled? How long will follow-up last? How will the playing field be kept level? In the event of medical complications, will the prescribing physician be deemed medically negligent? What is the funding model for the care of athletes participating in TEG? There are numerous other unresolved questions that pose significant challenges and threaten the foundational principles of TEG.

### Prevalence of Doping

An assumed high prevalence of doping has been cited in promotional materials for TEG, insinuating that a large proportion of athletes are already using PEDs in a clandestine way to avoid detection [6]. Notably, doping prevalence in sports with a high aerobic demand usually includes the use of erythropoietin (EPO), strength-oriented sports rely on anabolic steroids (AS) and combat sports tend to use stimulants for explosive energy boosts [7–9]. Diuretics and masking agents appear frequently in the test results of all sports, suggesting attempts to hide PED use [6]. While the available data suggest that most athletes competing in sport events under WADA rules do not use prohibited substances and methods, some numbers are a cause for concern. Research published in 2011 by members of the IAAF’s (now World Athletics (WA)), medical commission revealed potentially alarming prevalence of doping with microdoses of EPO, ranging from 54-99% in males and 35-68% in females for the same country, and for another country ranging from 21-87% in males and 5-66% in females [9]. This research comes after the launch in 2009 of the athlete biological passport (ABP), WADA’s main detection method for blood doping [10]. WADA soon thereafter developed a survey-based method to assess doping prevalence in elite sport using an indirect estimation model, which includes random elements to safeguard individual responses to sensitive questions. Using this approach, prevalence of past-year doping at the 13th World Championships in Athletics and at the 12th Pan-Arab Games was between 21 and 44% and between 11 and 57%, respectively [11, 12]. While acknowledging the difficulties in precisely determining doping prevalence in elite sport, a more recent study estimated a blood doping prevalence of 18% in the 2011 and 15% in the 2013 WA World Championships and attributed the decrease in prevalence to the implementation of the ABP [13]. Comparatively, the reporting of less than 1% of adverse analytical findings disclosed by WADA suggests a significant gap between the rate of detected cases (doping incidences) and the rate of admitted or estimated use (doping prevalence).

### Elite Sport: A Paradox of Health and Risk Even without PEDs

The adverse health effects of PED use are greatly underappreciated, threaten athletes’ careers, and pose additional risks after retirement [14]. Without full knowledge of the short- and long-term adverse effects, athletes are not well positioned to find the balance between performance and long-term well-being. Researchers have already described the direct medical consequences of PED use, including a wide variety of musculoskeletal, cardiovascular, endocrine and neurologic disorders. In addition, PEDs can impair immunity and even increase the risk of death [14]. However, the extent and mechanism of how PEDs exert adverse health effects remains speculative.

It is widely accepted that participation in elite sport is generally favourable to longevity, with longer life expectancy [15], lower all-cause mortality and reduced cardiovascular mortality [16] compared with the general population and attributed to the combination of genetic profiles and structured lifestyles. However, the risk of injury, residual impairment and even long-term disability represents significant adverse outcomes. For example, in a cross-sectional survey conducted with athletes who participated in Olympics between London 1948 and PyeongChang 2018, two-thirds of Olympians reported at least one Olympic-career significant injury and one-third of retired Olympians attributed functional limitations to their careers [17]. International Olympic Committee (IOC) in-games surveillance studies reported that up to 10% of athletes sustained injuries and 4% experienced illnesses during the Tokyo 2020 Summer Olympics [18]. Of the reported injuries, 56% did not result in time loss from sport [18]. During the Beijing 2022 Winter Olympics, the injury rate was higher, with 14.4% of athletes sustaining injuries and 4.6% experiencing illnesses [19]. The higher injury rates during the Winter Olympics reflect the inherent risks of winter sports, such as alpine skiing and snowboard slopestyle, which involve high-speed and aerial manoeuvres. Additionally, the closed-loop environment implemented at the Beijing Games to mitigate coronavirus disease 2019 (COVID-19) may have influenced illness rates [19]. In today’s world, sport has evolved into an industry and a source of national pride, which may contribute to the use of PEDs, as human performance with training alone cannot be improved endlessly [20].

### Goldman’s Dilemma and Modern Interpretation

Currently, the willingness of athletes to risk mortality for success has diminished significantly in sport culture – from over 50% previously to less than 1% [21]. A poll of 198 aspiring Olympians mostly from the United States (US), including sprinters, swimmers, powerlifters, and others, revealed that more than 50% athletes would be willing to take drugs that guarantee success but will lead to death within 5 years [22]. These data underscore the extreme lengths to which athletes may go when motivated by the pressures of competition, fame, and financial rewards, and suggest the desire to win may outweigh concerns for long-term well-being. A reformulation of Goldman’s Dilemma, where 211 athletes from various competitive levels (international, national, and regional championships) yielded only 1% acceptance of this lethal trade-off under the same hypothetical scenario presented in the original survey [23].

However, given that real-world doping decisions are made on the basis of the expectation that the benefits will outweigh the risks [23], the original Goldman question was unable to capture the sentiments of athletes considering PEDs with realistic health consequences. Therefore, this recent study further elicited athletes’ willingness to accept a PED associated with the risk of a fatal event instead of certain death, reflecting the estimated risk tolerance of the respondents [23]. When the drug was described as legal but with the same fatal outcome, a slightly higher cumulative percentage of participants (6 out of 109) indicated a willingness to take the risk. This suggests that legality may influence decision-making, even in the face of similar risks. Notably, around 12% of participants would dope when assured there would be no health consequences regardless of the legality of the substances [23]. This result is comparable to results from previous prevalence studies [13, 24], highlighting a persistent willingness of some athletes to engage in risky behaviours for competitive advantage. The study [23] provides valuable insights for policymakers, anti-doping agencies and sport organisations in promoting clean competition. Although Goldman’s dilemma may no longer be relevant to today’s elite athletes, it is essential to acknowledge the performance narrative in elite sport, which values single-minded dedication to the sport and prioritises winning above all other aspects of life [25].

### The First Declaration on Human Enhancement

Some people feel the health risks related to PEDs are more likely due to limited access to knowledgeable healthcare practitioners and the use of PEDs from black-market sources [26]. Hence, the First Declaration on Human Enhancement was launched in December 2024 aiming to explore health in a world of enhancement; Article 2 of this declaration [27] emphasises that the health and well-being of individuals take precedence over the right to enhancement. This may imply that individuals have the right to seek appropriate medical supervision while undergoing any form of enhancement. However, little is known about the willingness of professionals, aside from the Medical and Scientific Committee of TEG, to assist with enhancement. Furthermore, WADA prohibits medical professionals working with athletes who compete under the WADA Code from assisting and/or providing care to athletes using banned substances and methods, making it impossible from a legal perspective to ensure long-term athlete well-being while pursuing enhancement [28]. Additionally, the declaration mandates that event organisers are obligated to provide appropriate medical oversight and continuously improve access to it for individuals seeking or undergoing any form of enhancement [27]. Nonetheless, the concept of appropriate medical supervision can often be vague; standard medical supervision does not adequately address the need for identifying those at greater risk of serious adverse effects, making it difficult to ensure that all athletes receive the tailored support they need.

“Bodily autonomy” was introduced at the First Conference on Human Enhancement to maximise options for scientific innovation within a structural protection model. This concept evolved in the declaration as “The Right to Take Risk”, which enables individuals to take proportional risk in an informed manner and free from infringement by any institution [27]. However, this can lead to an expected conflict between athlete safety and autonomy. People who consider the use of PEDs and banned methods may overlook the long-term consequences or may be misinformed, distorting their ability to assess risks rationally. Moreover, a wide variety of stressors, such as the pursuit of fame and fortune, could compel athletes to accept risks they had not anticipated. In addition, the declaration states scientists have the duty to safely extend the limits of human potential through innovation, science and medicine; urges competition organisers to make all decisions on a scientific basis; and entitles stakeholders to knowledge regarding the role of enhancements in sport and society [27]. Urging competition organisers to make all decisions on the basis of scientific evidence assumes that there is a clear and universally accepted body of knowledge. Scientific research is contentious, and interpretations may vary, leading to decisions that are not necessarily in the best interest for athletes. The assertion of expanding limits potentially undermines the goals of athletes’ well-being. In the absence of systematic scientific evidence on the health effects of PEDs, obsessively pursing enhancements will bring athletes back into the Goldman’s dilemma.

Although TEG promise long-term assessment and continuous medical supervision, limited literature on TEG underscores the complex interplay between innovation, ethical challenges and potential transformations in competitive sport. Critics emphasise inherent health risks, ambiguities surrounding sport governance, safety protocols and the potential for adverse societal impacts [30, 31]. Moreover, as Klesney [29] highlights through Schumpeter’s perspective, innovation divorced from its ethical and moral foundations—when pursued solely for efficiency—can contribute to systemic instability, underscoring the vital interplay between economic progress and moral responsibility. The IOC condemned TEG, stating they contradict the values of the Olympics by undermining fair play [32]. Similarly, WADA has labelled the concept “dangerous and irresponsible”, emphasising the health risks to athletes. National anti-doping agencies have also voiced their disapproval. For example, UK Anti-Doping has reaffirmed its stance against doping, rejecting any place for such substances in sport [33]. Other national Olympic committees, including the Australian Olympic Committee and the Swedish Olympic Committee, have denounced the idea as reckless and contradictory to the spirit of sport [34]. Sport federations, such as World Athletics, echo this sentiment, with President Sebastian Coe dismissing TEG as lacking credibility and seriousness [35].

As with the abovementioned organisations, the International Federation of Sports Medicine/Fédération Internationale de Médecine du Sport (FIMS) remains committed to preserving the integrity of sport and protecting athletes from the risks associated with PEDs and has taken a clear stance against TEG. In their position paper [3], FIMS disputes TEG’s claims of prioritising safety through medical screening, arguing that such measures are insufficient to detect the long-term health risks associated with PED use. Furthermore, the paper warns of exacerbating inequalities in resource access, particularly disadvantaging athletes from less affluent nations, and highlights the potential exploitation of young, aspiring athletes. However, despite its opposition, FIMS acknowledges the potential for TEG to advance doping science by openly studying PED use in a safer and legal framework. Such research could improve detection methods, enhance athlete education on health risks and potentially deter PED use by exposing its long-term consequences.

As TEG continues to evolve, it provides a platform for significant debate and an opportunity to reconsider the trajectory of competitive sport. As such, proponents [36–38] highlight TEG’s potential benefits, including fostering inclusivity, destigmatising the use of image-enhancing drugs and PEDs, promoting harm reduction and offering new perspectives on fairness and athlete welfare. Proponents argue that TEG could complement existing frameworks, with Cox and Piatkowski [36] drawing parallels to bodybuilding’s tested and untested divisions, envisioning a model where TEG and the traditional Olympics coexist. This dual approach, they suggest, could offer athletes and spectators distinct paradigms while providing a unique opportunity to conduct research in an environment where PED use is allowed and controlled. Ekdahl and Krieger [39] expand on TEG’s broader ambitions, including economic and ecological sustainability, “enhanced athlete” representation and inclusivity. They position TEG as a potential testing ground for new sporting paradigms that challenge the established Olympic model, although they caution against unresolved concerns about biotechnological transformations of sport and the specifics of TEG’s implementation plans.

## Aims

The future of TEG is contingent upon five interrelated factors: (i) the participation of a sufficient number of top-level athletes; (ii) support from an athlete’s entourage, including coaches, sport physicians, physiotherapists, psychologists and their agents, peers, friends and family; (iii) a safe legal framework; (iv) sustainable financial backing; and (v) widespread public interest and acceptance. In these respects, TEG seems to emulate the established model of competitive sport. A significant concern around TEG is the currently limited perspectives from sport medicine professionals on the implications of TEG for athlete care, ethical considerations and the management of health risks. Given the notable health risks that TEG will pose on “enhanced athletes” and the significant responsibility that medical teams will assume, this study aims to address this gap by exploring the perspectives of sport physicians and other healthcare professionals regarding their potential roles in treating and supporting “enhanced athletes”.

## Methods

### Study Design

This study employed a self-report survey design to collect primary data from sport physicians and other allied healthcare professionals. This method, which is widely used in health and social care research, enabled the efficient and expedient gathering of data directly from professionals worldwide.

### Measures

Data were collected anonymously via a brief online survey with six closed questions using dichotomous (yes/no) response options, supplemented by one professional involvement question which offered four choices (orthopaedic and trauma surgeons, sport medicine specialists or internal medicine specialists; sport scientists; sport physiotherapists and similar health professionals; and other).

The questions addressed two main areas: (i) demographic information, including profession, past and present experiences in caring for professional athletes and team sport players and managing acute injuries, trauma or illnesses and (ii) willingness to treat “enhanced athletes” in various scenarios, as well as their readiness to support such athletes in using substances for performance enhancement. It is important to note that the questions were presumed to pertain to “enhanced athletes” only and were not extended to those competing under WADA regulations. Under the WADA Code, physicians who support athletes in using prohibited substances or methods risk facing significant professional, legal and personal consequences.

The survey is presented in Appendix 1. The survey was only available in English, which was considered sufficient given the participants’ international level of involvement in sport, where English is the dominant working language.

### Participants

Physicians, sport scientists and allied healthcare professionals from national/international sport medicine federations and organisations were invited to take part.

### Data Collection

The survey was accessible via a secure online platform. Participants were invited via email and snowballing (i.e. asking participants to share with others with similar profile) through personal contacts within sport medicine communities. Data were collected over a 2-month period. Geographical information was recorded on the basis of the IP addresses. Completion of the survey took less than 2 min.

### Data Analysis

Descriptive statistics were presented as counts and percentages for professional roles, experience with professional athletes and survey responses, primarily focusing on the graded willingness of treating “enhanced athletes” among the study participants. Results were limited to describing the sample without generalising the outcomes from this study. Chi-squared tests of association were used for group comparisons between “orthopaedic and trauma surgeons/sport medicine specialists/internal medicine specialists”, referred to here as “medical professionals” (MPs) and “sport scientists/sport physiotherapists and similar health professionals, along with other”, referred to here as “performance and rehabilitation experts” (PREs). Responses were analysed using Excel™ and RStudio™.

## Results

A total of 323 participants contributed their opinions. The majority (81.4%) of the respondents were sport physicians, followed by other allied health professionals (7.4%), sport scientists (5.9%) and sport physiotherapists and similar healthcare professionals (5.3%).

**Fig. 1:**
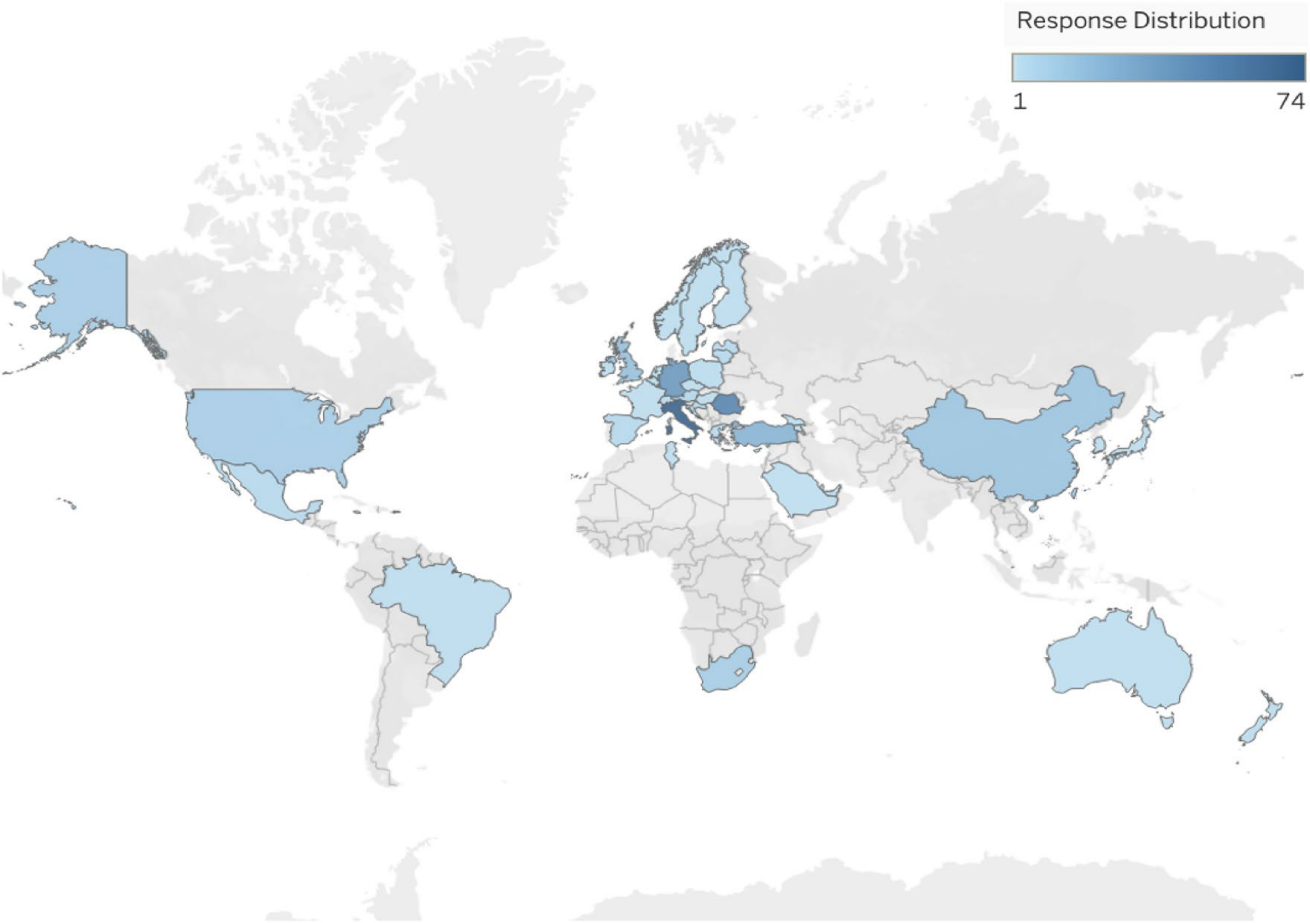
Country distribution of participants

Participants submitted responses from 46 countries and regions, with Italy (22.3%), Romania (16.4%) and Germany (12.7%) being at the top three with the highest number of participants (Fig. 1).

Among all the responses, 263 (81.4%) indicated having experience in treating professional athletes, 238 (73.7%) reported experience in treating athletes in team sport and 269 (83.3%) had treated acute lesions or chronic diseases in their careers.

### Willingness to Treat and Support “Enhanced Athletes”

After considering the “enhanced” background of athletes, willingness to treat decreased, with 237 (73%) participants willing to treat athletes with acute lesions and 233 (72%) willing to treat those with chronic diseases. However, 96 respondents (30%) indicated that they would be willing to help an athlete “enhance” their levels/support with medications knowing that they are an “enhanced athlete”, with the understanding that a safe legal framework would be provided, whereas 225 (70%) rejected the idea. Three participants (0.9%) did not answer the questions about treating or supporting “enhanced athletes”.

### Differences by Professional Role, Experience and Country of Residence

Among those who expressed willingness to support “enhanced athletes”, 28 countries and regions were recorded (Table 1). The geographical distribution of respondents who expressed hypothetical support for an “enhanced athlete” using medication to enhance their levels or support proportionally reflected the broader respondent base, indicating no significant regional variation in willingness to support “enhanced athletes”. However, individual professional numbers are not representative of their countries and region.

**Table 1:** Countries and regions expressed willingness to support “enhanced athletes”

Countries and regions	Number of respondents	Percentage (%) willing to support enhanced athletes
Armenia	1	1.04%
Austria	3	3.12%
China	3	3.12%
Czech Republic	1	1.04%
Germany	11	11.46%
Spain	2	2.08%
UK	7	7.29%
Greece	2	2.08%
Hong Kong SAR	1	1.04%
Croatia	2	2.08%
Hungary	1	1.04%
Ireland	1	1.04%
Italy	22	22.91%
Jamaica	1	1.04%
Japan	1	1.04%
Liechtenstein	1	1.04%
Lithuania	2	2.08%
Luxembourg	1	1.04%
Latvia	2	2.08%
Malta	1	1.04%
Mexico	1	1.04%
Netherlands	2	2.08%
Puerto Rico	1	1.04%
Romania	9	9.38%
Turkey	12	12.50%
United Arab Emirates	1	1.04%
USA	2	2.08%
South Africa	2	2.08%
Total	96	100.00%

SRA, special administrative region

**Fig. 2:**
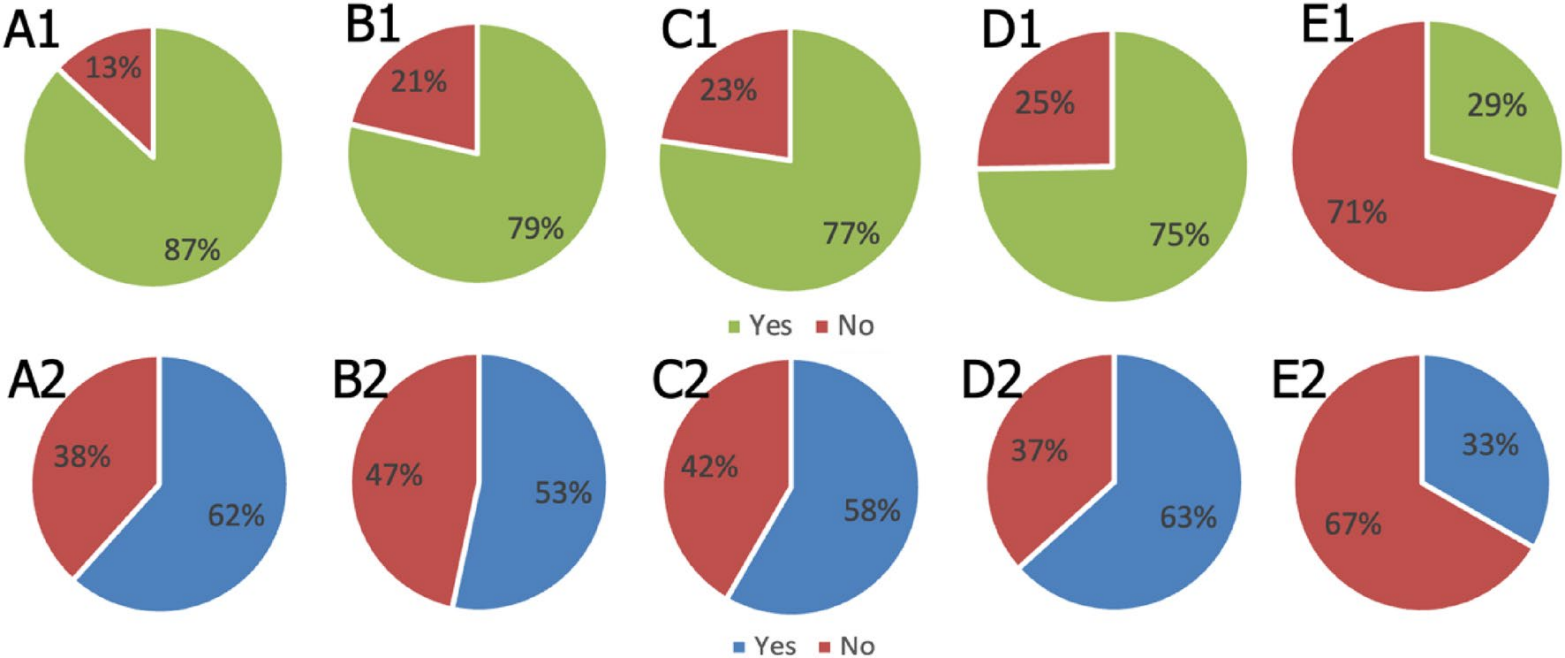
Voting results from MPs: A1) Are you treating professional athletes in your office/hospital/site? B1) Are you involved in team sports treatments? C1) Would you treat an athlete in your field with acute lesion knowing that he/she is an “enhanced athlete”? D1) Would you treat an athlete in your field with chronic diseases knowing that he/she is an “enhanced athlete”? E1) Would you help an athlete to “enhance” their levels/support with medications knowing that he/she is an “enhanced athlete”? Same questions apply to PREs in A2) B2) C2) D2) E2)

MPs and PREs expressed different levels of willingness to treat and/or supporting “enhanced athletes” (Fig. 2). A total of 263 participants, of whom 87% had experience in treating professional athletes and 79% team sport experience, belonged to the MPs category. In contrast, among the 60 PREs, only 62% had worked with professional athletes, and 53% had experience in team sport. Statistically significant differences were observed in the willingness towards treating acute conditions between the two groups, with MPs demonstrating a higher willingness than PREs (77% MPs versus 58% PREs; *p* < 0.01). The readiness to treat chronic conditions was statistically similar, with 75% of MPs and 63% of PREs expressing a willingness to treat individuals with chronic conditions (*p* = 0.11). Furthermore, their responses on refusing to assist an athlete in “enhancing” their performance through medications showed a high degree of consistency, with 71% of MPs and 67% of PREs sharing this stance (*p* = 0.64).

## Discussion

The debate surrounding TEG and the use of PEDs highlights a critical juncture in elite sport. While TEG advocates for controlled and transparent use of PEDs to advance science and fairness, their stance challenges the long-established principles of the IOC and WADA. Both organisations have condemned TEG’s approach, asserting that it violates the ethical, legal and health standards central to global sport. This makes it difficult for sport physicians to reconcile their commitment to treat all patients regardless of their beliefs and values. This was reflected in our preliminary results showing that sport medicine professionals are prepared to treat acute injuries or chronic diseases in “enhanced athletes”—possibly seeing them first as patients, though their choices maybe disagreeable. However, fewer of them were willing to assist athletes with aspects such as access, prescriptions and dosing. Although those who expressed willingness to support athletes are in the minority, the proportion of such sport medicine professionals is not irrelevant; our results suggest that nearly one in three sport physicians and aligned healthcare providers could be open to working with athletes on their “pharmacological training”, given that a safe legal framework is provided. This emphasises the necessity of implementing comprehensive and legal support systems aimed at safeguarding the short- and long-term well-being of athletes.

The willingness of sport medicine professionals to treat injuries or illnesses in athletes known to be enhancing provides valuable insights into their ethical and professional judgments. Akin to treating AS users among bodybuilders, this willingness represents an altruistic commitment to providing care for human beings, regardless of their enhancement choices. It is essential to emphasise, however, that approximately 30% of respondents indicated a reluctance to provide care to such athletes. This raises an important question: Would these professionals truly refuse care to an “enhanced athlete” in a real-world scenario, especially if faced with the immediate ethical challenge of providing necessary medical assistance? Who regulates and controls the use of forbidden substances in young athletes on their way to elite level? This is still an ongoing discussion. It is plausible that, in practice, the refusal rate might be significantly lower, as professional responsibility and ethical duty would outweigh personal or ideological reservations.

Scholars examining TEG often draw upon literature on PED use in bodybuilding and harm reduction strategies [30, 37, 38]. Against this backdrop, the findings of our study are particularly noteworthy. The broad support for providing care to “enhanced athletes” and the notable willingness among some physicians to collaborate with these athletes in their pursuit of pushing human boundaries contrast with prior research on physicians’ openness and preparedness to care for AS users [41–43]. The latter may be partly due to the limited specialised knowledge among physicians regarding AS use in general healthcare settings [42] and partly due to the mutual mistrust between users and healthcare professionals documented in earlier studies [43]. Instead, community pharmacists have been suggested as a potential resource for advice and harm reduction for AS users [44]. Pharmacists are often the first point of contact for athletes seeking information on drug dosing and side effects. With their expertise in pharmacology, they are well-positioned to provide trusted and confidential advice on the safe use of medications, educate athletes on potential adverse effects and implement harm-minimisation interventions in community pharmacy settings. The role of pharmacists in anti-doping efforts has also been highlighted in recent years [45–47], particularly at the sub-elite level, where athletes often have limited or no access to sport physicians. In many countries, pharmacists already play a key role in delivering harm-minimisation interventions in the community pharmacy setting; they manage needle and syringe exchange programmes, provide specific sterile equipment for injecting AS, offer information on safe injection techniques and infection prevention measures and establish referral links to other medical and rehabilitation services [48]. For example, in Japan, a national programme has existed since 2009 to train and register pharmacists as “sport pharmacists”. This initiative aims to help athletes make safe and informed decisions about self-medication and provides education on anti-doping issues within the community pharmacy setting [49]. This programme exemplifies how pharmacists could be more widely empowered to play a front-line role in delivering both trusted advice and harm-minimisation interventions to athletes. Pharmacists could therefore play an increasingly important role in bridging gaps in care and harm reduction for “enhanced athletes”.

## Clinical, Research and Policy Implications

To help athletes achieve peak performance while protecting their health, only enhancements that are scientifically proved, legal and consistent with the principles of the Hippocratic Oath [40] should be permitted, where the tenet “I will do no harm or injustice to them. Neither will I administer a poison to anybody when asked to do so, nor will I suggest such a course” must be strictly followed. Appropriate care of athletes undergoing enhancements will also require athletes to fully disclose all modes of enhancements to their healthcare providers so that any interactions/side effects can be closely monitored. Furthermore, the willingness of sport medicine professionals to treat acute injuries and chronic diseases, as well as to support enhancements, is grounded in robust clinical data regarding how PEDs affect athletes’ health and well-being. As new research findings emerge and a safer framework is adapted, the opinions of healthcare providers regarding the treatment of enhanced athletes are likely to evolve.

To advance our understanding of PEDs, both in terms of performance-enhancing potential and health risks [14], future studies should be conducted with elite athletes to address the current knowledge gap in the field of PEDs. First, epidemiologic surveys should be performed to determine the real prevalence of PED use among elite athletes. These surveys will provide insights into the current use of PEDs in elite sport, which in turn will inform anti-doping policies and strategies. Second, while the effects of some widely abused substances on performance are well-documented, the existence of residual physiological effects remains unclear. Therefore, it is crucial to undertake observational cohort studies to determine the long-term health effects, with the aim of exploring not only the physiological impacts but also the psychological and social consequences of substance use, providing a comprehensive understanding of how these factors influence athlete well-being over time. Even for the known complications of PEDs, there have been no randomised trials of therapies. Therefore, it is important to conduct randomised trials to identify optimal therapeutic strategies, especially for preventing and treating AS withdrawal syndrome. Leveraging artificial intelligence (AI)-driven gene sequencing could further enhance our understanding of individual responses to treatment and help tailor therapeutic approaches for those experiencing withdrawal.

Moreover, studies to identify mechanisms and biomarkers related to PEDs should be performed in animals in parallel with humans. Previous mouse models and observational human studies on AS use have proposed the muscle memory mechanism elaborating its long-lasting effects on muscle performance [41]. Utilising multi-omics solutions, which include transcriptomics, proteomics, metabolomics and epigenomics, will provide a deeper understanding of the molecular mechanisms associated with androgenic steroid-induced muscle memory. Expanding the biobank in terms of muscle, plasma, saliva and urine samples combined with longitudinal intervention study designs [e.g. EPO, testosterone, human growth hormone (HGH)] allows researchers to examine the utility and sensitivity of new biomarkers to be identified from hypothesis-free multi-omics studies.

## Limitations

While the pilot survey provides valuable insights into healthcare professionals’ attitudes towards supporting “enhanced athletes”, it faces several methodological limitations, which primary lie in the relatively small sample size from the major Olympic sport nations as well as international federations, and the lack of representation from countries is also prominent, especially those where doping is a persistent concern. The survey’s dichotomous questions, not allowing for open-ended responses, could oversimplify participants’ views and their ethical stances. The English-only format may have resulted in a significant cultural and linguistic barrier that reduced the validity of the findings. Moreover, the reliance on IP address information to determine respondents’ countries of residence may have introduce some degree of inaccuracy because participants could have completed the survey while on holiday, while traveling or while using a virtual private network (VPN), which could route their IP address through a different country. Additionally, the study focused exclusively on the perspectives of one key stakeholder group—sport physicians and allied health professionals—and explores their views within a hypothetical framework. As with the Goldman dilemma, the gap between responses to a hypothetical scenario and real-life decision-making could be substantial. This potential discrepancy underscores a limitation in interpreting the survey findings, which should be viewed as a tentative response to an undefined hypothetical scenario. The ambiguity arises from the limited understanding at this stage of what an “enhanced athlete” would entail in terms of capabilities and the legal conditions under which they would operate.

WADA and other relevant organisations believe that support personnel who assist athletes in participating in TEG could potentially be at risk of committing anti-doping rule violations under the Code. Furthermore, physicians should be aware that providing PEDs without medical need could put them at risk, e.g., their medical licensure, and even in criminal jeopardy in some countries. This danger certainly will influence physicians’ willingness to care for or support “enhanced athletes” using WADA-prohibited substances and/or methods in practice.

The limitations of the present study, particularly the fact that the survey captures a single moment in time, must be addressed in future research. Opinions and attitudes expressed by respondents may evolve as new data, societal pressures or regulatory changes emerge. For instance, future developments in performance-enhancing technologies, shifts in public opinion or changing legal and ethical standards could significantly influence the willingness of sport medicine professionals to engage with “enhanced athletes”. Acknowledging this limitation underscores the need for ongoing research to track these attitudes over time and to contextualise findings within the dynamic environment of elite sport. By exploring these issues, this study not only contributes to the literature on sport medicine and enhancement but also opens new avenues for dialogue about the role of healthcare professionals in supporting athlete health and well-being in increasingly complex competitive landscapes.

## Recommendations for Future Research

Building on the findings of the present study, a more comprehensive and inclusive survey targeting a broader group of healthcare professionals together with elaboration of a safe legal framework is highly necessary. Employing stratified sampling methods to capture diverse subgroups of healthcare professionals (e.g. sport physicians, physiotherapists, pharmacists, and psychologists) would further enrich the findings and allow for more targeted analysis of stakeholder-specific perspectives. Providing the survey in multiple languages would enhance accessibility and inclusivity and yield a more globally representative dataset. Particular attention should be given to including participants from countries with limited access to anti-doping resources and those operating under differing legal and cultural frameworks regarding the use of PEDs.

To capture the complexity of perspectives, the survey design should incorporate a variety of question formats that allow for more nuanced and detailed responses. For example, graded scaling (Likert scales or other graded measures to assess the intensity of respondents’ view and beliefs), vignettes with hypothetical scenarios illustrating different aspects of treating or supporting “enhanced athletes”, which can help contextualise and clarify decision-making processes; or best–worst scaling, which forces respondents to prioritise their preferences, would generate data for deeper insights into the factors influencing the judgments of sport physicians and other healthcare professionals regarding caring for and/or supporting “enhanced athletes”. Open-ended questions can elicit qualitative data for the exploration of the underlying reasons, ethical considerations and contextual factors shaping respondents’ views. Qualitative exploration of the reasons and rationalisations behind sport physicians’ stance regarding treating versus aiding “enhanced athletes” would greatly enhance our understanding of the challenges and the opportunities TEG presents for healthcare professionals working with elite athletes within legal and ethical limits.

## Conclusions

While TEG positions itself as a revolutionary alternative to anti-doping policies, questions and concerns about the long-term health implications and the ethics of performance enhancement persist, as shown by our survey of MPs and PREs—a key group of stakeholders in this conversation. The recently published Declaration on Human Enhancement, which prioritises the health of the athletes over enhancement, and their commitment to promote only safe enhancements are a welcome start. As the dialogue on human enhancement evolves, it is essential to ensure that the safety of athletes remains a non-negotiable fundamental principle.

## Appendix 1

Questionnaire.

## Abbreviations

ABP: Athlete biological passport
AS: Anabolic steroid
EPO: Erythropoietin
FIMS: International Federation of Sports Medicine
IOC: International Olympic Committee
MPs: Medical professionals
PREs: Performance and rehabilitation experts
PEDs: Performance-enhancing drugs
TEG: The Enhanced Games
WA: World Athletics
WADA: World Anti-Doping Agency

## Declarations

### Data Availability

The data supporting this study, which include de-identified data, are available upon reasonable request. Interested researchers can obtain the data by contacting the corresponding author (Y.P.) at ypitsiladis@hkbu.edu.hk.
